# Supplementary figures and images for: Evidence from studies in rodents and in isolated adipocytes that agonists of the chemerin receptor CMKLR1 may be beneficial in the treatment of type 2 diabetes
Source: PeerJ. 2015 Feb 5;3:e753. doi: 10.7717/peerj.753 (PMC4327305; doi:10.7717/peerj.753)

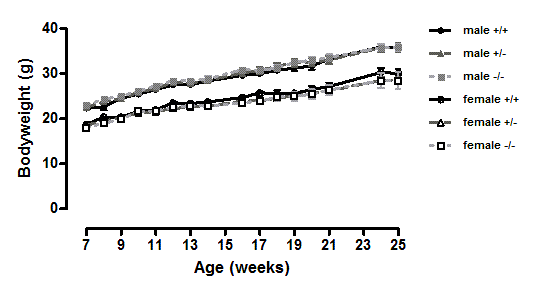

Supplement: Figure S1 — n = 15 for wild-type and heterozygote mice, and 11 and 7 for male and female knockout mice respectively. The upper three lines are for males and the lower three for females. [file peerj-03-753-s001.png]

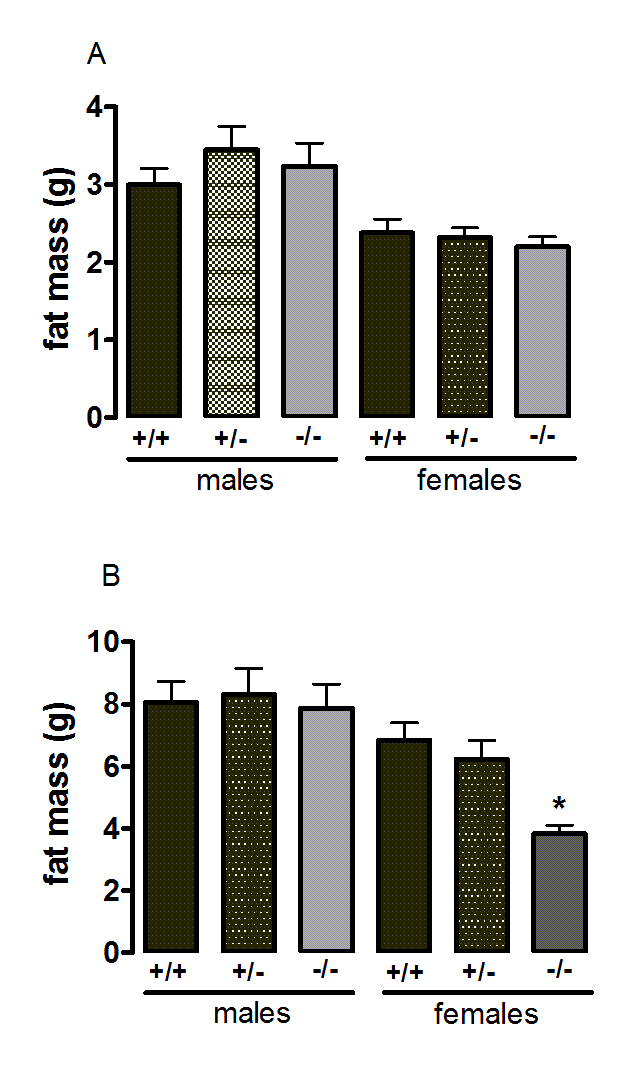

Supplement: Figure S2 — n values are as for supplementary Fig. 1. [file peerj-03-753-s002.png]

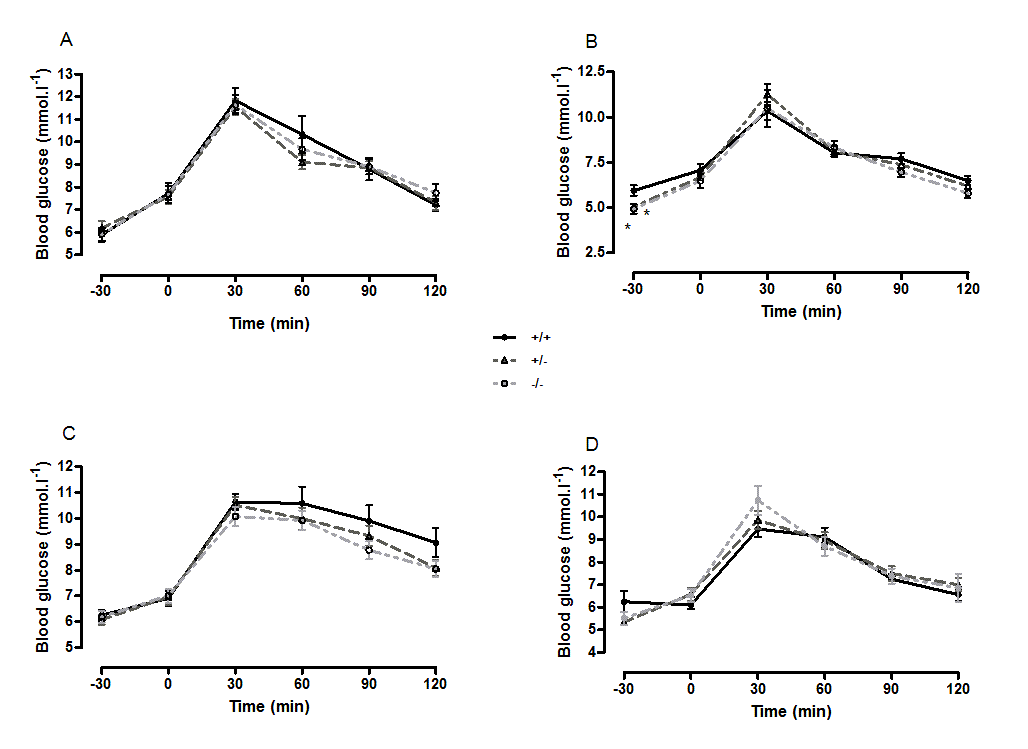

Supplement: Figure S3 — n values are as for Supplementary Fig. 1. ∗P < 0.05 for knockout and heterozygote compared to wild-type mice of the same sex. [file peerj-03-753-s003.png]

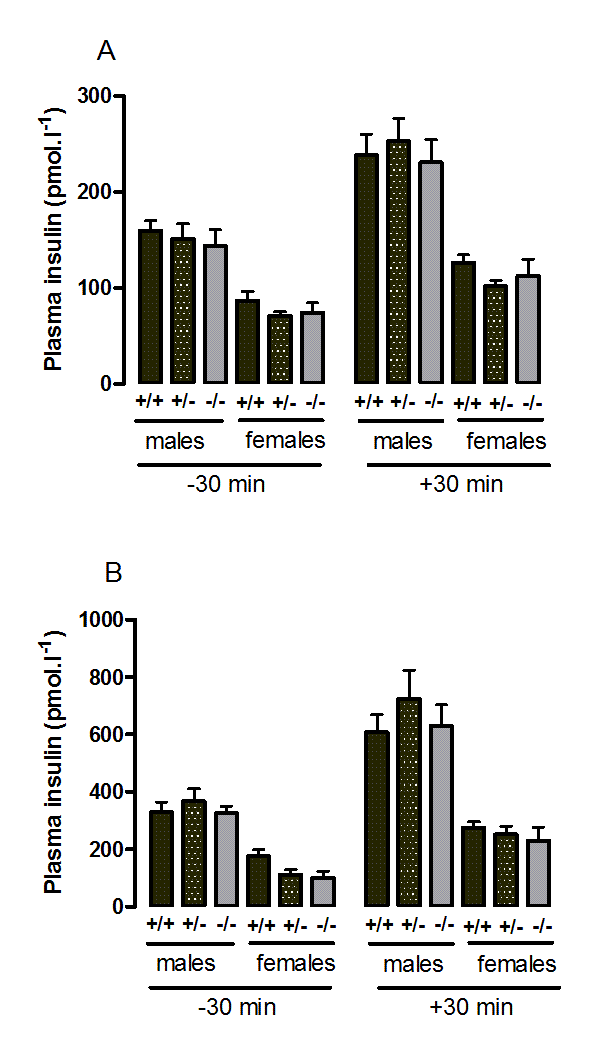

Supplement: Figure S4 — n values are as for Supplementary figure 1. [file peerj-03-753-s004.png]

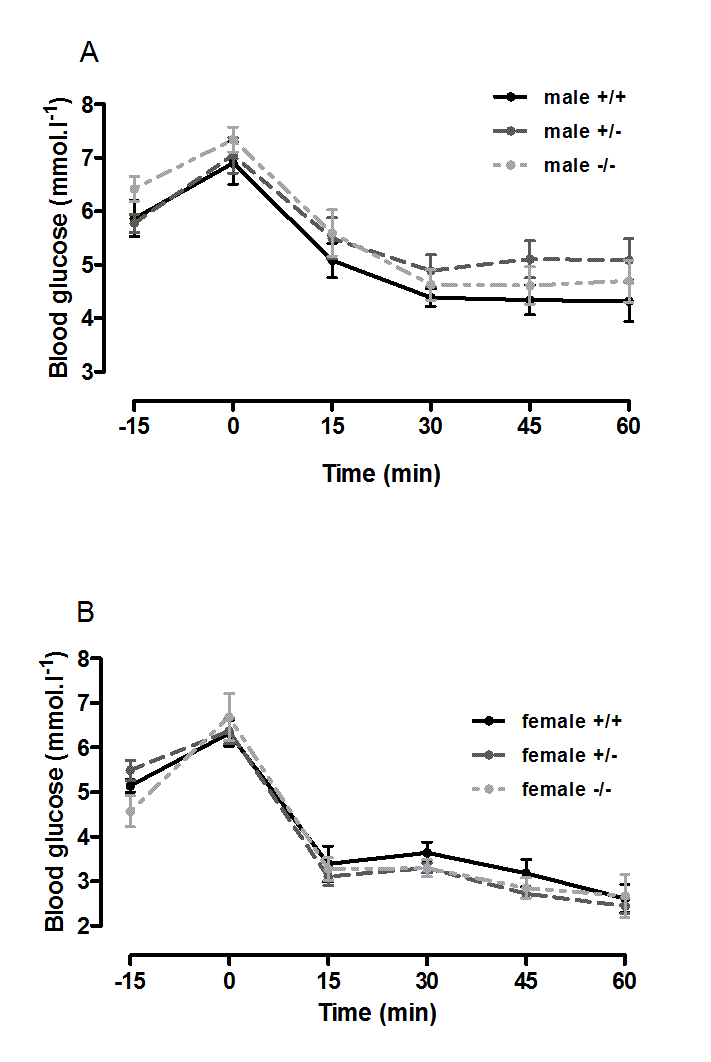

Supplement: Figure S5 — n values are as for Supplementary figure 1. [file peerj-03-753-s005.png]

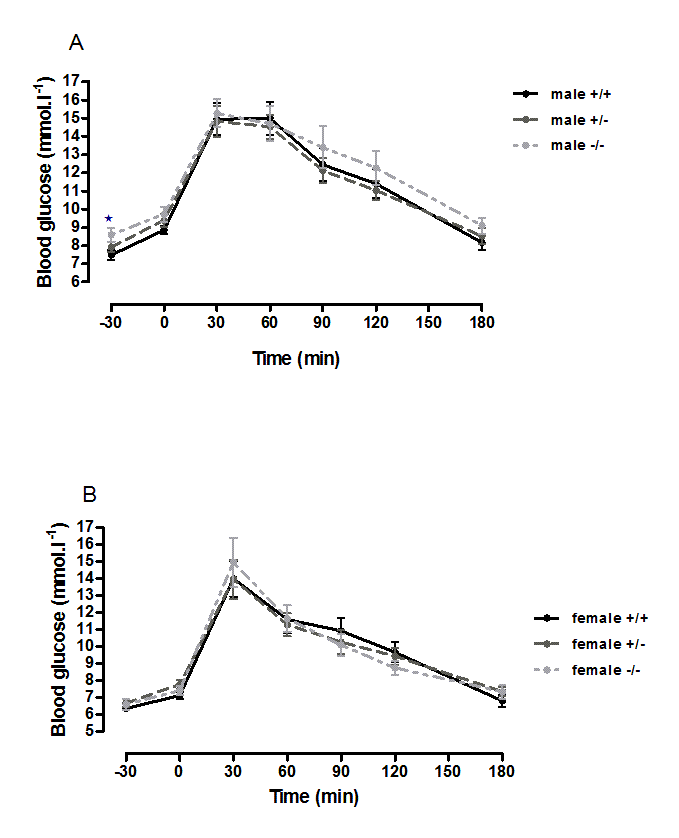

Supplement: Figure S6 — n = 17 to 21, except for female knockout mice, where n = 10. [file peerj-03-753-s006.png]

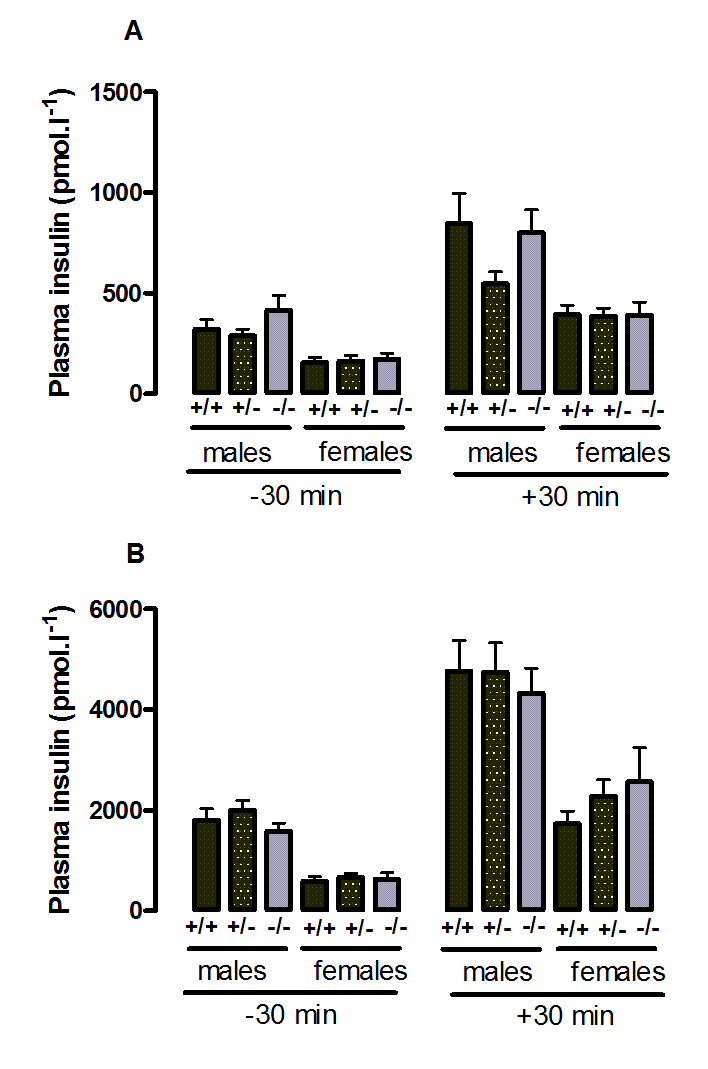

Supplement: Figure S7 [file peerj-03-753-s007.png]

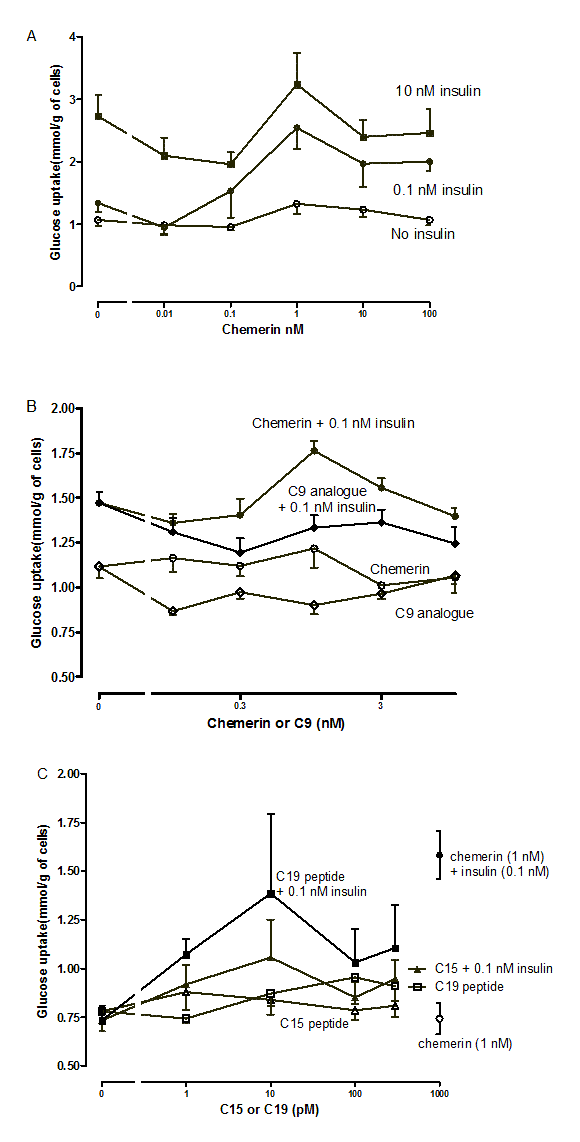

Supplement: Figure S8 — Panel (A) shows consolidated results for two preparations of adipocytes which gave very similar basal values and where concentrations of chemerin were identical between experiments; n = 4 to 10. Panels(B) and (C) show means for 4 replicates from single preparations of adipocytes. [file peerj-03-753-s008.png]

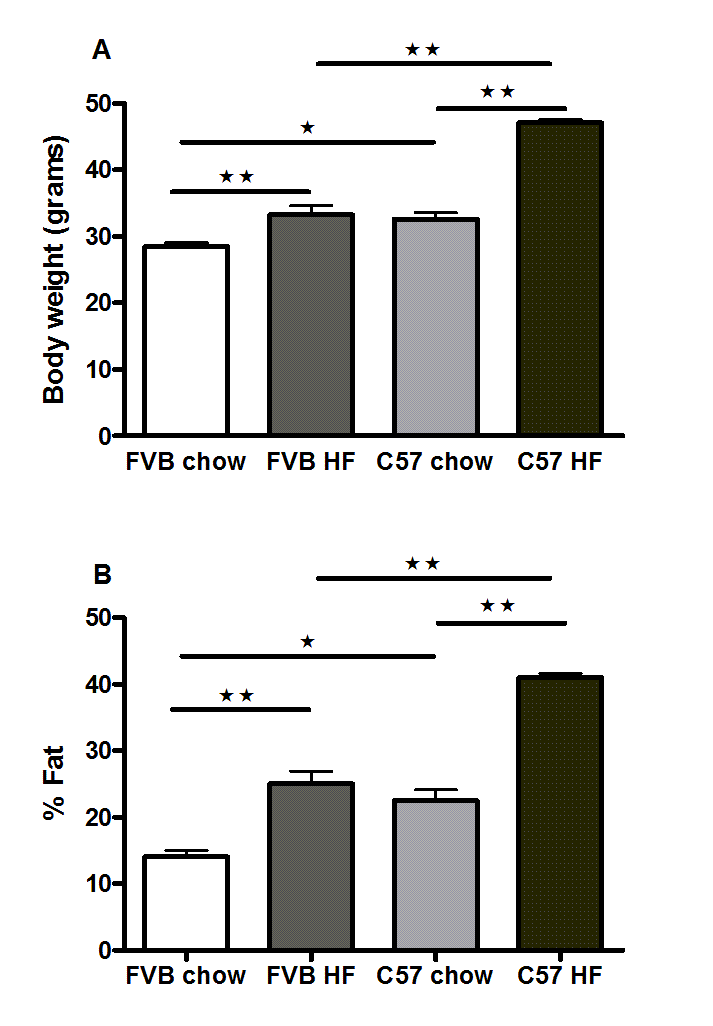

Supplement: Figure S9 — n = 7 to 9. ∗P < 0.05; ∗∗P < 0.01; ∗∗∗P < 0.001 for the comparisons indicated by the bars. [file peerj-03-753-s009.png]

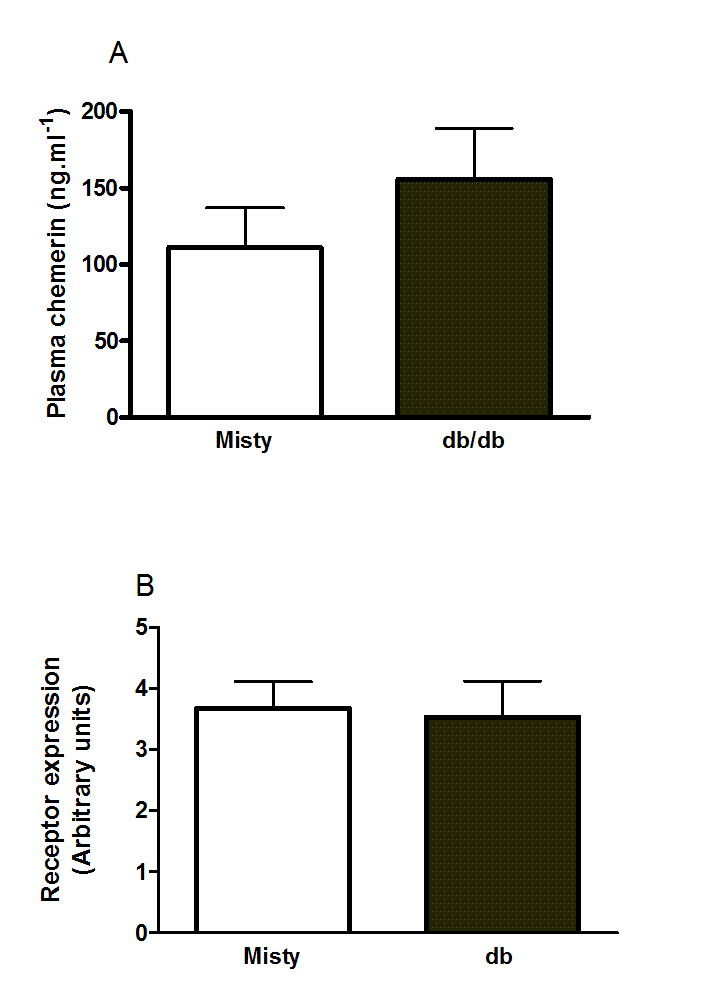

Supplement: Figure S10 — n = 6 in A and 5 in B. [file peerj-03-753-s010.png]

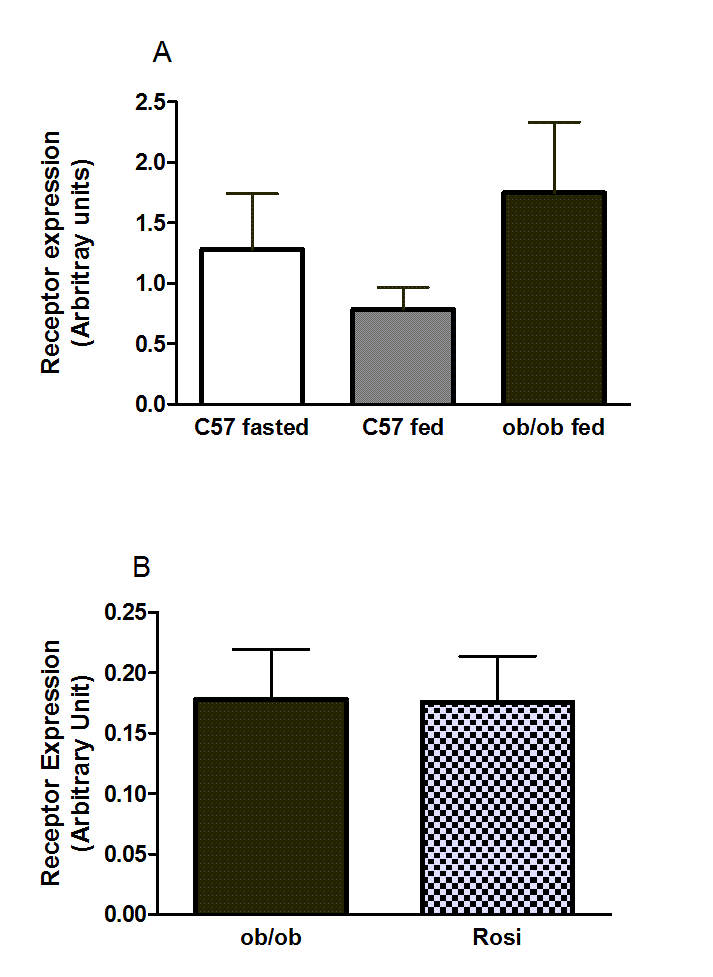

Supplement: Figure S11 — (A) Fed and 5 h-fasted C57BL/6 8-week-old wild-type and 10-week-old ob/ob mice, and (B) female ob/ob mice treated with rosiglitazone for 3 weeks. n values and other details are given in the legends to Fig. 7D and 8B of the main paper, which, by contrast, show changes in RARRES2 expression in inguinal fat. [file peerj-03-753-s011.png]
